# Dietary patterns and risk of breast cancer

**DOI:** 10.1038/sj.bjc.6606044

**Published:** 2010-12-14

**Authors:** L Baglietto, K Krishnan, G Severi, A Hodge, M Brinkman, D R English, C McLean, J L Hopper, G G Giles

**Affiliations:** 1 Cancer Epidemiology Centre, The Cancer Council Victoria, 100 Drummond Street, Carlton, Melbourne, Victoria 3053, Australia; 2 Centre for Molecular, Environmental, Genetic and Analytical Epidemiology, University of Melbourne, Melbourne, Australia; 3 Department of Medicine, St Vincent's Hospital, University of Melbourne, Melbourne, Australia; 4 The Alfred Hospital, Melbourne, Australia; 5 Department of Epidemiology and Preventive Medicine, Monash University, Melbourne, Australia

**Keywords:** breast cancer, dietary patterns, factor analysis, prospective study

## Abstract

**Background:**

Evidence is emerging that prudent/healthy dietary patterns might be associated with a reduced risk of breast cancer.

**Methods:**

Using data from the prospective Melbourne Collaborative Cohort Study, we applied principal factor analysis to 124 foods and beverages to identify dietary patterns and estimated their association with breast cancer risk overall and by tumour characteristics using Cox regression.

**Results:**

During an average of 14.1 years of follow-up of 20 967 women participants, 815 invasive breast cancers were diagnosed. Among the four dietary factors that we identified, only that characterised by high consumption of fruit and salad was associated with a reduced risk, with stronger associations observed for tumours not expressing oestrogen (ER) and progesterone receptors (PR). Compared with women in the lowest quintile of the factor score, the hazard ratio for women in the highest quintile was 0.92 (95% confidence interval (CI)=0.70–1.21; test for trend, *P*=0.5) for ER-positive or PR-positive tumours and 0.48 (95% CI=0.26–0.86; test for trend, *P*=0.002) for ER-negative and PR-negative tumours (test for homogeneity, *P*=0.01).

**Conclusion:**

Our study provides additional support for the hypothesis that a dietary pattern rich in fruit and salad might protect against invasive breast cancer and that the effect might be stronger for ER- and PR-negative tumours.

Most of the studies on the risk of breast cancer in relation to individual foods and nutrients have had inconsistent results (Michels *et al*, 2007). Dietary pattern analysis is an increasingly common alternative approach for examining the relationship between diet and the risk of disease or biomarkers of disease (Newby and Tucker, 2004). Factor analysis applied to dietary data produces a few patterns of dietary intake that represent underlying independent dimensions of food and nutrient consumption. The assessment of dietary patterns in this way overcomes limitations of the single food or nutrient approach (Newby and Tucker, 2004). Two recent systematic literature reviews (Edefonti *et al*, 2009; Brennan *et al*, 2010) found consistent evidence that a ‘prudent’/‘healthy’ dietary pattern was associated with a reduced risk of breast cancer.

Breast cancer, similar to most cancers, is heterogeneous in its pathology, natural history and response to treatment. Breast cancer differ, for example, with respect to oestrogen (ER) and progesterone receptor (PR) status; hormone receptor-positive cancers exhibiting better differentiated morphological appearance and stronger clinical response to hormonal treatment (Althuis *et al*, 2004). There is evidence to suggest that risk factors (Colditz *et al*, 2004), including food items (Olsen *et al*, 2003; Stripp *et al*, 2003; Suzuki *et al*, 2005; Zhang *et al*, 2005; Cho *et al*, 2006) and dietary patterns (Fung *et al*, 2005; Velie *et al*, 2005; Agurs-Collins *et al*, 2009) differ in their association by tumour receptor status.

Using data from the Melbourne Collaborative Cohort Study (MCCS), we analyze the effect of dietary patterns on breast cancer risk overall, by attained age during follow-up and by tumour characteristics, including tumour grade and ER and PR status.

## Materials and methods

The MCCS is a prospective cohort study of 41 514 people (24 469 women) aged 27–76 years at baseline (99.3% of whom were aged 40–69), recruited in 1990–1994 in the Melbourne metropolitan area. Subjects were recruited via the Electoral Rolls (registration to vote is compulsory in Australia), advertisements and community announcements in local media (e.g. television, radio and newspapers). The Cancer Council Victoria's Human Research Ethics Committee approved the study protocol. Subjects gave written consent to participate and for the investigators to obtain access to their medical records.

At baseline interview, questions were asked about conventional risk factors, such as reproductive history, hormone replacement therapy and oral contraceptive use, country of birth, alcohol consumption, physical activity, smoking habits and highest level of education. Subjects also completed a dietary questionnaire that included questions relating to dietary habits and a 121-item food frequency questionnaire (FFQ), specifically developed for the MCCS (Ireland *et al*, 1994). Intake of energy was computed using Australian food composition tables (Lewis *et al*, 1995), and included energy from the FFQ and energy from alcohol. Standard procedures were used to measure height and weight at baseline attendance for each participant (Lohman *et al*, 1988; MacInnis *et al*, 2004a) from which body mass index (BMI) was calculated.

Women were excluded from the study if they did not complete the dietary questionnaire (*N*=11); reported extreme values of total energy intake (<1st percentile or >99th percentile) (*N*=488) or a history of angina, diabetes or heart disease before baseline as their diet might have changed in consequence (*N*=1421); had a confirmed diagnosis of invasive breast cancer before baseline (*N*=415); or had missing values in any of the potential confounders (*N*=1167). These exclusions left 20 967 women eligible for these analyses.

Cases included women with a first diagnosis of adenocarcinoma of the breast (International Classification of Diseases for Oncology, 3rd edition, 10th revision rubric C50.0–C50.9) during follow-up until 31 December 2007. Cases were ascertained by record linkage to the population-based Victorian Cancer Registry (VCR), which covers the state in which the cohort resides, and to the National Cancer Statistics Clearing House, which holds cancer incidence data from all Australian states. Women with *in situ* breast cancer were not counted as cases. Addresses and vital status of all subjects were determined by record linkage to Electoral Rolls, Victorian death records, the National Death Index, from electronic phone books and from responses to mailed questionnaires and newsletters.

The medical records of women with breast cancer were reviewed and their cancers classified according to tumour grade and ER and PR status as recorded in histopathology reports held at the VCR. We repeated the measurement of ER and PR status for a subset of cases with archival tissue available (67% of all cases in the sample). The original diagnostic tumour slides for these cases were retrieved from pathology laboratories and reviewed by a single pathologist (CM) who assessed ER and PR status using immunohistochemistry techniques. The archival material was sectioned at four micron and placed on superfrost plus slides. A routine dewaxing procedure was followed by heat-induced epitope retrieval with either citrate buffer pH 6 or TRIS EDTA pH 8 using a DAKO (Carpinteria, CA, USA) Pascal pressure chamber. The following antibodies were used: ER (Labvision rabbit monoclonal SP1) at 1/250, PR (DAKO PGR636) at 1/1200. Immunoreaction was performed using a Lab Vision (Fremont, CA, USA) autostainer using Lab Vision HRP polymer detection system and DAKO DAB+. The agreements between the ER and PR status assessed by immunohistochemistry and the values held by the VCR were 89 and 81%, respectively (for ER, *κ*=0.71, *P*<0.001; for PR, *κ*=0.62, *P*<0.001). Because of the good agreement between the ER and PR data, when archival tumour tissue was not available, ER and PR status was assigned according to the histopathology reports held at the VCR.

### Statistical analysis

Factor analysis was performed on the 121 items from the FFQ, plus olive and vegetable oil and alcohol from wine (other alcoholic beverages were rarely consumed by women). For the 121 items, intake was measured as daily equivalent frequency, intake of oils was measured as ml per week, and alcohol as g per day. The principal factor method was used to extract factors, followed by orthogonal (varimax) rotation to assist in interpretation of the factors and to ensure that the factors were uncorrelated. Factors with Eigenvalues of 2 or greater were retained. Variables with factor loadings having absolute values of 0.2 or greater were used in interpreting the factors. For each rotated factor, scores were computed as the sum of products of the standardized observed variables multiplied by weights proportional to the factor loadings. Scores were categorized in quintiles.

Multivariate regression methods were used to calculate how much of the variance in factor scores was associated with country of birth, total energy intake and potential confounders.

Follow-up began at baseline and continued until diagnosis of breast cancer (*N*=815), diagnosis of unknown primary cases (*N*=49), death (*N*=1216), date left the area covered by the cancer registries (*N*=117) or 31 December 2007, whichever came first. Hazard ratios (HRs) were estimated using Cox regression with age as the time metric. Analyses were adjusted for potential confounders, including country of birth, total energy intake, age at menarche, duration of lactation, parity (parous *vs* nulliparous), oral contraceptive use (ever *vs* never), hormone replacement therapy (never, past and current users), menopausal status at baseline, level of education (some primary education, some secondary education and completed secondary education, degree or diploma), level of physical activity (none, low, medium and high; see MacInnis *et al*, 2004b for further detail), total alcohol intake (lifetime abstainers, ex-drinkers, 1–19 g per day, 20 g per day or more), smoking and BMI. In order to account for the heterogeneity of the association with BMI by menopausal status, we treated BMI as an age-dependent variable, by splitting the data into two age groups (⩽55 and >55) and including an interaction between BMI (continuous) and age groups. We estimated separate HRs for the two follow-up age groups (⩽55 and >55), by fitting Cox models with the inclusion of a term for the interaction between factor scores and groups, and for each dietary factor score overall and by grade and hormone receptor status of the tumours. To test for heterogeneity in the HRs by grade (well *vs* moderately *vs* poorly differentiated), and ER and PR status (ER− *vs* ER+ PR− *vs* PR+ and ER+ or PR+ *vs* ER− and PR−), Cox's proportional regression models were fitted using a data duplication method (Lunn and McNeil, 1995). For sensitivity analysis purposes, we repeated all analyses (i) including only women born in Australia/New Zealand/the United Kingdom, (ii) excluding the first two years of follow-up, (iii) censoring after 10 years of follow-up, (iv) using ER and PR measures from immunohistochemistry only or the VCR only. To address the possibility that differential reporting of alcohol intake according to dietary patterns biased the associations, the analysis of risk overall was stratified by alcohol drinking after excluding ex-drinkers.

Tests for linear trend were based on pseudo-continuous variables under the assumption that all subjects within each quintile of factor score had the same score, equal to the within-quintile median. Statistical analyses were performed using Stata 11.0 (Stata Corporation, College Station, TX, USA).

## Results

Table 1 summarizes the baseline characteristics. In total, 79% of the women were born in Australia, New Zealand or in the United Kingdom and 21% in Italy or Greece. The mean age at baseline was 55 (range: 31–76 years) years; 34% of the women were aged under 50 years, 17% between 50 and 55 years and 49% over 55 years. We identified 815 incident invasive breast cancer cases (813 were histopathologically verified) over an average of 14.1 years of follow-up between baseline attendance and 31 December 2007 for the eligible 20 967 women.

The mean age at diagnosis of cases was 63 years (range: 41–85 years), 22% were diagnosed before age 55 years, 12% within the first 2 years of follow-up and 30% after 10 years. The status of ER or PR was known for 95% of the cases, of which 575 (74%) were ER+ and 202 (26%) ER− and 426 (55%) were PR+ and 349 (45%) PR−. There were 605 (78%) cases expressing ER or PR and 173 (22%) cases expressing neither ER nor PR. Information on grade was available for 741 (91%) cases, including 162 (22%) well-differentiated, 333 (45%) moderately differentiated and 246 (33%) poorly differentiated tumors. Tumours not expressing hormone receptors had a higher grade than those expressing ER or PR (76% of the ER− and PR− tumours and 22% of the ER+ or PR+ tumours were poorly differentiated).

Four factors with eigenvalues greater than 2 were identified that explained 66% of the variance in all the food and beverage items. Rotated factor loadings for food items with an absolute value of 0.2 or greater for any factor are reported in Table 2. The four factors were characterized as follows; (1) high intakes of vegetables, boiled rice, wholemeal bread, yoghurt, chicken, fish (not fried), potato cooked without fat, fruit salad, banana and pineapple, with low intakes of white bread; (2) high intakes of salad greens, cucumber and fruit; (3) high intakes of desserts, cheddar cheese, margarine, lamb, sausages, bacon, potato cooked without fat, green beans and peas, pumpkin, tea, chocolate, other confectionary, jam, honey and vegemite, with low intakes of olive oil, pasta or noodles, ricotta and feta cheese, beef or veal schnitzel, steamed fish, legume soup, tomato, salad vegetables, legumes, olives and figs; (4) high intakes of fried rice, white bread, pizza, savoury pastries, feta cheese, fried eggs and egg dishes, meats (fresh and processed), fried fish, pickled vegetables, potatoes cooked in fat and olives.

Correlations with energy intake were 0.36, 0.27, 0.35 and 0.44 for factors 1–4, respectively (Table 3). Factor 1 had a strong positive correlation with vegetable consumption (*r*=0.87), which indicates that higher intake of the diet pattern characterized by factor 1 is associated with higher intake of vegetables, and is labelled ‘vegetable’. Factor 2 had a strong positive correlation with fruit (*r*=0.90); because it was also characterized by high consumption of salad green, it is labelled ‘fruit and salad’. Factor 3 was positively correlated with cereals (*r*=0.36), dairy products (*r*=0.30), margarine (*r*=0.43) and potatoes (*r*=0.37) and negatively correlated with olive oil (*r*=−0.43) and leafy green vegetables (*r*=−0.37); because women born in Australia or New Zealand scored higher on this factor (Table 4) it is labelled ‘traditional Australian’. Factor 4 was mainly positively correlated with red meat (*r*=0.75) and is labelled ‘meat’.

Of all the variables listed in Table 1, only country of birth, level of education and total energy intake explained more than 5% of the variance in any factor, with country of birth explaining between 5 and 34% of the total variance of the factors (Table 4). Women born in Italy or Greece had higher scores for the ‘fruit and salad’ and ‘meat’ patterns and women born in Australia or New Zealand had higher scores for the traditional Australian pattern; level of education was positively associated with the ‘vegetable’ pattern and negatively associated with the ‘meat’ pattern.

Scores for the ‘fruit and salad’ pattern were inversely associated with breast cancer risk (HR for the highest *vs* the lowest quintile of the factor, 0.81; 95% confidence interval (CI): 0.63–1.03; test for trend, *P*=0.03; Table 5). There was a marginal statistically significant association between the ‘traditional Australian’ pattern and breast cancer risk before the age of 55 years (Table 5). No other dietary pattern was significantly associated with breast cancer risk (Table 5). There was no evidence of heterogeneity by attained age for the association between risk and any of the dietary patterns (Table 5).

Table 6 shows the association between the four dietary pattern scores and breast cancer risk by tumour ER and PR status. The ‘fruit and salad’ pattern score was inversely associated with the risk of ER-negative (HR for the highest *vs* the lowest quintile of the factor, 0.55; 95% CI: 0.32–0.93; test for trend, *P*=0.004; test for homogeneity *P*=0.03) and PR-negative breast cancer (HR for the highest *vs* the lowest quintile of the factor, 0.67; 95% CI: 0.46–0.98; test for trend, *P*=0.01; test for homogeneity *P*=0.08), whereas no association was observed for ER-positive or PR-positive cancers (Table 6). When cases were classified according to their ER and PR status combined, the HR for the highest *vs* lowest quintile of the ‘fruit and salad’ pattern were 0.92 (95% CI: 0.70–1.21; test for trend, *P*=0.5) for ER+ or PR+ tumours and 0.48 (95% CI: 0.26–0.86; test for trend, *P*=0.002) for ER− and PR− tumours (test for homogeneity by ER/PR, *P*=0.01).

Scores for the ‘fruit and salad’ pattern were inversely associated with high-grade tumours, (HR for the highest *vs* the lowest quintile, 0.58; 95% CI: 0.36–0.92; test for trend, *P*=0.001), whereas no association was observed for moderate or low-grade tumours (test for homogeneity by grade, *P*=0.02). None of the other three patterns showed heterogeneity by tumour grade (results not shown).

Similar results were obtained when the analyses were restricted to women born in Australia/New Zealand/the United Kingdom or when excluding the first 2 years of follow-up, or when censoring after 10 years of follow-up or when the analyses by tumour ER and PR status used the data from our own immunohistochemistry only or the data from histopathology reports held by the VCR only (not shown).

There was no evidence of heterogeneity of the association between dietary pattern and breast cancer risk by level of alcohol consumption (not shown).

## Discussion

Using data from a prospective cohort study of 20 967 women with an average follow up of 14 years we identified four dietary patterns that we described as ‘vegetable’, ‘fruit and salad’, ‘traditional Australian’ and ‘meat’. Overall, we observed an inverse association between breast cancer risk and the ‘fruit and salad’ dietary pattern. This inverse association was more pronounced for hormone receptor-negative tumours and for high-grade tumours. No other dietary pattern was associated with risk.

The MCCS is a multicultural cohort that includes a high proportion of Southern European migrants in order to extend the range of lifestyle exposure, including diet. It has long and virtually complete follow-up of participants and has extensive data on potential confounding variables. Baseline diet was measured using an FFQ specifically designed to capture the variety of food consumption. To avoid making assumptions about food consumption patterns, we chose not to group the food items for the dietary factor analysis (Hodge *et al*, 2007). This allowed us to identify four dietary patterns that together reflected the usual dietary intake of our population. The factors were correlated with, but not synonymous with country of birth, as shown by the relatively low proportion of the variance in factors’ scores explained by country of birth, and by the fact that all associations with dietary patterns were confirmed when restricting the analysis to women born in Australian, New Zealand and the United Kingdom. Ascertainment of cases through linkage with the VCR provided us with accurate information about grade and hormone receptor status for a high proportion of the tumours. We found good agreement between the ER and PR status of the tumours recorded on histopathology reports held by the VCR and those obtained from immunohistochemical anlaysis of archival tissue using a standardised protocol.

Excluding the first 2 years of follow-up did not change the results, suggesting that changes in diet due to preclinical lesions are unlikely to have contributed to the observed associations.

A limitation of this study is the single measure of diet. Diet was recorded only once at baseline using a FFQ that only showed a fair to moderate agreement for foods when administered on two occasions 12 months apart (Hodge *et al*, 2007). We cannot exclude that random error in measuring dietary intake and dietary change during follow-up might have attenuated any association between dietary patterns and breast cancer risk. Our data do not provide evidence of heterogeneity of the associations by alcohol intake. This suggests that it is unlikely that the observed associations are due to differential reporting of alcohol consumption in the different categories of dietary pattern. However, we cannot exclude that the positive finding are due to residual confounding or to the chance given the number of comparisons we performed.

The association between fruit and vegetables and breast cancer is biologically plausible because of their high contents of potentially anticarcinogenic compounds (Collins, 2005), but epidemiological studies of individual foods or of food groups indicate that high intakes of fruit or vegetables have a limited or no major effect on breast cancer incidence (Smith-Warner *et al*, 2001b; IARC, 2003; Willett, 2005; van Gils *et al*, 2005; AICR, 2007). On the other hand, the literature on dietary patterns and breast cancer risk suggests that a diet characterized by food with high fat and high sugar content (western dietary pattern) is associated with an increased risk of breast caner, whereas a diet characterized by vegetables, fruit, fish and white meat (prudent dietary pattern) is associated with a reduced risk (Edefonti *et al*, 2009; Brennan *et al*, 2010). Between the two ‘prudent’ patterns we identified, one characterized by high intake of vegetables and the other by high intake of fruits and salad, only the latter was associated with reduced breast cancer risk. Fruit and vegetables both contain high levels of nutrients with antioxidant properties, such as carotenoids and vitamins (Steinmetz and Potter, 1991). Some of these nutrients are destroyed by cooking, such as oxygenated carotenoids (e.g., lutein), the predominant carotenoid of green leafy vegetables (spinach, broccoli, Brussels sprouts and cabbage), water-soluble vitamin C, and folate (Steinmetz and Potter, 1991) and this might explain why we found a protective effect against aggressive breast cancers for the fruit and salad pattern, characterised by high intake of fruit and salad, but not for the vegetable pattern, characterised by high intake of vegetables that are usually consumed cooked. The evidence on the role of single vitamins in the risk of breast cancer is inconsistent (Zhang, 2004), but there is some evidence suggesting a role of multivitamin intake in decreasing the risk of ER-negative cancers, which might be due to folate (Ishitani *et al*, 2008). The Nurses’ Health Study found an inverse association between total folate intake and ER-negative tumours, that could be explained by the role of folate in maintaining normal DNA methylation; aberrant DNA methylation might be associated with the loss of ER expression (Zhang *et al*, 2005). In our study, we found that the inverse association between with the ‘fruit and salad’ pattern was stronger for ER- or PR-negative tumours. The literature reporting associations of risk with fruits and vegetables by hormonal receptor status is sparse and inconsistent (Olsen *et al*, 2003; Gaudet *et al*, 2004; Fung *et al*, 2005; Sant *et al*, 2007; Cui *et al*, 2008; Agurs-Collins *et al*, 2009). Similar to our finding, results from the Danish Diet, Cancer and Health cohort (Olsen *et al*, 2003), the Nurse's Health study (Fung *et al*, 2005) and the Black Women's Health Study (Agurs-Collins *et al*, 2009) suggest that a prudent diet and higher intakes of fruits and vegetables might protect against ER-negative tumours, but not against ER-positive tumours. In the ORDET study, the salad and oil pattern was particularly associated with HER-2-positive cancers (Sant *et al*, 2007) and HER-2 over-expression is associated with more aggressive and ER-negative cancers. It has been suggested that for breast cancers that are less dependent on hormones, such as ER− and PR− breast cancers, the protective effect of phytochemicals present in fruit and vegetables on many cellular functions may be fully expressed, whereas for ER+ and PR+ cancers it can be overridden by hormonal factors (Olsen *et al*, 2003; Fung *et al*, 2005). More aggressive breast cancers, and tumours not expressing ER or PR receptors only represent a small proportion of all breast tumours and epidemiological studies considering breast cancer as a single homogeneous disease might have failed to detect any effect due to the inclusion of a small number of aggressive tumours not expressing hormone receptors.

We did not find significant evidence of an association with the other two dietary patterns that we identified, the meat pattern, correlated with high consumption of meat, and the traditional Australian pattern, correlated with high consumption of dairy products and margarine. Studies of dietary fat intake and risk have been inconsistent (Howe *et al*, 1990; Hunter *et al*, 1996; Smith-Warner *et al*, 2001a; Boyd *et al*, 2003; Cho *et al*, 2003; Thiebaut *et al*, 2007). Evidence from the Nurse's Health Study II also suggest that higher red meat intake may be a risk factor for premenopausal ER+/PR+ breast cancer (Cho *et al*, 2006). We had too few data to perform the analysis by age and hormone receptor status.

Our analysis supports the hypothesis that a dietary pattern characterized by high intake of fruit and salad is associated with a slightly lower risk of breast cancer and for hormone receptor-negative breast cancer in particular. Breast cancer is a heterogeneous disease and we have provided additional evidence that risk factors might differ in their relationship to cancer subtypes.

## Figures and Tables

**Table 1:** Characteristics of the study population

	**All women (*N*=20 967)**	**BC cases (*N*=815)**
**Characteristic**	***N* (%)**	***N* (%)**
Age, mean (s.d.)	55 (9)	56 (8)
*Country of birth*		
Australia/New Zealand/Other	15 143 (72)	647 (79)
The United Kingdom	1391 (7)	40 (5)
Italy	2476 (12)	77 (9)
Greece	1957 (9)	51 (6)
		
*Age at menarche (years)*		
<12	3411 (16)	136 (17)
12	4113 (20)	149 (18)
13	5547 (26)	216 (27)
⩾14	7896 (38)	314 (39)
		
*Parity*		
Nulliparous	3085 (15)	147 (18)
Parous	17 882 (85)	668 (82)
		
*Duration of lactation (months)*		
Never	6177 (29)	274 (34)
Up to 6	4581 (22)	148 (18)
7–12	3321 (16)	114 (14)
13–24	4044 (19)	179 (22)
>24	2844 (14)	100 (12)
		
*Oral contraceptive*		
Never user	8378 (40)	329 (40)
Past user	12 187 (58)	472 (58)
Current user	402 (2)	14 (2)
		
*HRT*		
Never user	15 566 (74)	564 (69)
Past user	1774 (8)	67 (8)
Current user	3627 (17)	184 (23)
		
*Menopausal status*		
Having periods	7566 (36)	285 (35)
Periods stopped for natural reasons	8970 (43)	381 (47)
Periods stopped for other reasons^a^	4431 (21)	149 (18)
		
*Level of physical activity*		
None	4503 (21)	139 (17)
Low	4461 (21)	185 (23)
Medium	7599 (36)	317 (39)
High	4404 (21)	174 (21)
		
*Alcohol consumption*		
Lifetime abstainers	7866 (38)	298 (37)
Ex-drinkers	647 (3)	29 (4)
1–19 g per day	10 062 (48)	397 (49)
20–39 g per day	1850 (9)	66 (8)
40 g per day or more	542 (3)	25 (3)
		
*Smoking*		
Never	14 547 (69)	574 (70)
Current	1831 (9)	63 (8)
Former	4589 (22)	178 (22)
		
*Level of education*		
⩽Primary school	3902 (19)	114 (14)
Some high/technical school	9029 (43)	362 (44)
Completed high/technical school	3761 (18)	153 (19)
Degree/diploma	4275 (20)	186 (23)
		
BMI (kg m^–2^), mean (s.d.)	26.6 (4.8)	26.9 (4.9)
		
Energy from diet (Mj per day), mean (s.d.)	8.5 (2.8)	8.5 (2.8)

Abbreviations: BC=breast cancer; BMI=body mass index; HRT=hormone replacement therapy.

a Hysterectomy, ovariectomy or other reasons.

**Table 2:** Rotated factor loadings for food and beverage items with loadings having absolute values of 0.2 or greater for any factor

	**Rotated factor loading**			
**Food item**	**Factor 1**	**Factor 2**	**Factor 3**	**Factor 4**
Olive oil			−0.39	
Boiled rice	0.20			
Fried rice				0.28
White bread	−0.20			0.28
Wholemeal bread	0.25			
Sweet biscuits			0.33	
Cakes/sweet pastries			0.34	
Puddings			0.34	
Pasta/noodle dish			−0.25	
Pizza				0.23
Savoury pastries				0.32
Ricotta cheese			−0.27	
Fetta cheese			−0.26	0.25
Cheddar cheese			0.24	
Ice cream			0.25	
Custard			0.22	
Cream/sour cream			0.26	
Yoghurt	0.25			
Fried egg				0.27
Egg dish				0.24
Margarine			0.39	
Beef/veal schnitzel			−0.21	0.41
Beef/veal roast				0.44
Beef steak				0.32
Beef rissole				0.43
Beef dish				0.29
Roast/fried chicken				0.30
Boiled chicken	0.20			
Chicken dish	0.22			
Lamb roast/chops			0.28	0.23
Lamb dish				0.27
Pork roast/chops				0.25
Salami				0.21
Sausage/frankfurter			0.33	
Bacon			0.20	0.26
Steamed fish	0.24		−0.22	
Fried fish				0.34
Canned fish	0.22			
Legume soup			−0.25	
Pickled vegetables				0.21
Tomato	0.27		−0.23	
Capsicum	0.35		−0.28	
Salad greens	0.37	0.21	−0.32	
Cucumber	0.37	0.22	−0.35	
Celery/fennel	0.46		−0.21	
Beetroot	0.42			
Coleslaw	0.34			
Potato cooked in fat				0.36
Potato cooked without fat	0.35		0.33	
Carrot	0.56			
Cabbage/brussels sprouts	0.54			
Cauliflower	0.58			
Broccoli	0.58			
Leafy greens	0.45			
Green beans/peas	0.44		0.26	
Cooked dried legumes	0.29		−0.26	
Pumpkin	0.50		0.27	
Onion/leek	0.29			
Mushroom	0.27			
Sweet corn	0.23			
Zucchini/squash/eggplant	0.36			
Vegetable dish	0.25			
Fruit salad	0.21	0.21		
Orange/mandarin		0.43		
Apple		0.40		
Banana	0.25	0.25		
Peach/nectarine		0.66		
Pear		0.51		
Cantaloupe/honeydew		0.55		
Watermelon		0.54		
Strawberry		0.48		
Plum		0.65		
Apricot		0.67		
Pineapple	0.22	0.29		
Olives		0.28	−0.26	0.20
Fig		0.40	−0.20	
Grape		0.56		
Tea			0.39	
Chocolate			0.27	
Other confectionery			0.24	
Jam/honey			0.26	
Vegemite			0.22	

**Table 3:** Correlation between factors and energy intake and food groups

	**Vegetable**	**Fruit and salad**	**Traditional Australian**	**Meat**
Energy intake	0.36	0.27	0.35	0.44
				
*Food group*				
Olive oil	−0.10	0.12	−0.43	0.13
Vegetable oil	−0.06	0.02	−0.07	0.13
Cereals	0.19	0.07	0.36	0.11
Dairy	0.18	−0.01	0.30	0.04
Eggs	0.12	−0.01	0.10	0.34
Butter	0.02	−0.01	0.19	0.08
Margarine	0.12	−0.09	0.43	0.04
Red meat	0.05	0.03	0.17	0.75
Chicken	0.24	0.02	−0.13	0.35
Fish	0.31	0.04	−0.20	0.15
Vegetables	0.87	0.24	−0.26	0.14
Leafy green vegetables	0.54	0.24	−0.37	0.12
Potatoes	0.36	−0.02	0.37	0.14
Fruit	0.26	0.90	−0.02	−0.01

**Table 4:** Means (s.d.) of factor scores and *R*^2^ by categories of women's characteristics

	**Factors**			
	**Vegetable**	**Fruit and salad**	**Traditional Australian**	**Meat**
*Country of birth*				
Australia/New Zealand	0.12 (0.88)	−0.11 (0.81)	0.29 (0.77)	−0.14 (0.75)
The United Kingdom	0.14 (0.89)	−0.05 (0.87)	0.07 (0.71)	−0.09 (0.77)
Italy	−0.66 (0.75)	0.35 (1.16)	−0.91 (0.57)	0.17 (0.88)
Greece	−0.19 (1.15)	0.43 (1.14)	−1.12 (0.67)	0.94 (1.25)
*R* ^2^	0.08	0.05	0.34	0.13
				
*HRT*				
Never user	−0.04 (0.94)	0.01 (0.93)	−0.03 (0.92)	0.04 (0.91)
Past user	0.11 (0.91)	−0.03 (0.83)	0.11 (0.88)	−0.08 (0.80)
Current user	0.13 (0.87)	−0.03 (0.91)	0.09 (0.80)	−0.15 (0.77)
*R* ^2^	0.01	<0.01	<0.01	0.01
				
*Alcohol consumption*				
Lifetime abstainers	−0.05 (1.01)	0.08 (0.99)	−0.04 (1.03)	0.10 (0.99)
Ex-drinkers	0.04 (0.96)	0.01 (0.98)	−0.02 (0.93)	−0.09 (0.90)
1–19 g per day	0.03 (0.90)	−0.02 (0.88)	0.06 (0.82)	−0.07 (0.80)
20 g per day or more	0.03 (0.77)	−0.17 (0.78)	−0.09 (0.73)	0.00 (0.81)
*R* ^2^	<0.01	0.01	<0.01	0.01
				
*Level of education*				
⩽Primary school	−0.41 (0.98)	0.27 (1.06)	−0.82 (0.84)	0.48 (1.15)
Some high/technical school	0.05 (0.93)	−0.12 (0.86)	0.30 (0.82)	−0.06 (0.80)
Completed high/technical school	0.11 (0.85)	−0.05 (0.88)	0.13 (0.81)	−0.10 (0.77)
Degree/diploma	0.18 (0.84)	0.04 (0.90)	0.01 (0.72)	−0.22 (0.69)
*R* ^2^	0.05	0.02	0.21	0.07
				
*BMI*				
<25 kg m^–2^	0.09 (0.88)	−0.04 (0.87)	0.13 (0.81)	−0.17 (0.77)
25–29 kg m^–2^	−0.03 (0.93)	0.01 (0.95)	−0.03 (0.91)	0.05 (0.89)
30 kg m^–2^ or more	−0.13 (1.01)	0.07 (0.97)	−0.22 (1.00)	0.27 (1.00)
*R* ^2^	0.01	<0.01	0.02	0.04
				
*Total energy intake*				
I tertile	−0.37 (0.69)	−0.27 (0.62)	−0.34 (0.66)	−0.37 (0.55)
II tertile	0.00 (0.78)	−0.02 (0.83)	−0.02 (0.81)	−0.06 (0.69)
III tertile	0.37 (1.11)	0.29 (1.15)	0.36 (1.05)	0.43 (1.11)
*R* ^2^	0.13	0.07	0.12	0.19

Abbreviations: BMI=body mass index; HRT=hormone replacement therapy.

*R*^2^ measures how much of the variation in factor score is explained by each variable.

**Table 5:** Association between quintile of dietary pattern scores and breast cancer risk overall and by attained age during follow-up

		**Overall**		**By attained age during follow-up**				
				**⩽55 years**		**>55 years**		
**Dietary pattern**	**Quintiles**	**No. of cases**	**HR (95% CI)**	**No. of cases**	**HR (95% CI)**	**No. of cases**	**HR (95% CI)**	***P*-value** a
Vegetable	1	147	Reference	30	Reference	117	Reference	0.66
	2	154	0.97 (0.77, 1.22)	42	1.11 (0.69, 1.79)	112	0.93 (0.71, 1.21)	
	3	169	1.02 (0.81, 1.30)	39	1.07 (0.66, 1.74)	130	1.01 (0.77, 1.32)	
	4	185	1.10 (0.87, 1.40)	33	0.97 (0.58, 1.62)	152	1.13 (0.87, 1.48)	
	5	160	0.98 (0.76, 1.28)	36	1.23 (0.74, 2.05)	124	0.93 (0.69, 1.24)	
	*P*-trend		0.97		0.72		0.84	
Fruit and salad	1	174	Reference	47	Reference	127	Reference	0.47
	2	189	1.04 (0.84, 1.28)	41	0.88 (0.58, 1.34)	148	1.10 (0.86, 1.40)	
	3	158	0.87 (0.69, 1.08)	32	0.73 (0.46, 1.14)	126	0.91 (0.71, 1.18)	
	4	156	0.88 (0.70, 1.10)	33	0.73 (0.46, 1.15)	123	0.93 (0.72, 1.21)	
	5	138	0.81 (0.63, 1.03)	27	0.66 (0.40, 1.07)	111	0.86 (0.65, 1.13)	
	*P*-trend		0.03		0.10		0.11	
Traditional Australian	1	116	Reference	21	Reference	95	Reference	0.10
	2	163	1.34 (1.03, 1.76)	37	1.13 (0.65, 1.97)	126	1.41 (1.05, 1.91)	
	3	162	1.27 (0.94, 1.71)	41	1.28 (0.73, 2.22)	121	1.24 (0.89, 1.73)	
	4	202	1.54 (1.14, 2.09)	48	1.81 (1.05, 3.13)	154	1.44 (1.03, 2.01)	
	5	172	1.25 (0.90, 1.74)	33	1.58 (0.87, 2.85)	139	1.17 (0.82, 1.66)	
	*P*-trend		0.24		0.04		0.64	
Meat	1	162	Reference	25	Reference	137	Reference	0.16
	2	161	1.00 (0.80, 1.25)	30	1.08 (0.63, 1.85)	131	1.00 (0.78, 1.27)	
	3	168	1.07 (0.85, 1.34)	43	1.43 (0.86, 2.37)	125	1.00 (0.78, 1.29)	
	4	170	1.12 (0.88, 1.41)	38	1.23 (0.73, 2.07)	132	1.11 (0.86, 1.44)	
	5	154	1.12 (0.85, 1.46)	44	1.50 (0.88, 2.55)	110	1.03 (0.77, 1.38)	
	*P*-trend		0.45		0.11		0.93	

Abbreviations: BMI=body mass index; 95% CI=confidence interval; HR=hazard ratio; HRT=hormone replacement therapy.

Estimates from the model including four factor scores, adjusted for country of birth, age at menarche, parity, duration of lactation, oral contraceptive use, HRT use, menopausal status at baseline, physical activity, alcohol, smoking, level of education, total energy intake and BMI.

a Test for homogeneity by attained age at follow-up.

**Table 6:** Association between quintile of dietary pattern scores and breast cancer risk by ER and PR status of the tumor

		**By ER status**					**By PR status**				
		**Positive**		**Negative**			**Positive**		**Negative**		
**Dietary patterns**	**Quintiles**	**No. of cases**	**HR (95% CI)**	**No. of cases**	**HR (95% CI)**	***P*** a	**No. of cases**	**HR (95% CI)**	**No. of cases**	**HR (95% CI)**	***P*** a
Vegetable	1	101	Reference	35	Reference	0.41	82	Reference	53	Reference	0.39
	2	102	0.94 (0.70, 1.25)	43	1.02 (0.64, 1.63)		73	0.85 (0.61, 1.18)	72	1.14 (0.79, 1.64)	
	3	119	1.04 (0.78, 1.38)	43	0.99 (0.63, 1.58)		91	1.03 (0.74, 1.42)	71	1.05 (0.72, 1.52)	
	4	136	1.17 (0.88, 1.56)	42	0.94 (0.58, 1.51)		93	1.06 (0.76, 1.48)	84	1.19 (0.82, 1.71)	
	5	117	1.04 (0.75, 1.43)	39	0.92 (0.55, 1.55)		87	1.06 (0.74, 1.53)	69	0.98 (0.64, 1.48)	
	*P*-trend		0.6		0.53			0.47		0.61	
Fruit and salad	1	120	Reference	45	Reference	0.03	86	Reference	80	Reference	0.08
	2	127	1.01 (0.78, 1.30)	53	1.14 (0.76, 1.69)		89	0.99 (0.73, 1.34)	91	1.08 (0.80, 1.46)	
	3	111	0.87 (0.67, 1.14)	44	0.94 (0.61, 1.44)		88	0.98 (0.72, 1.33)	67	0.78 (0.56, 1.09)	
	4	111	0.91 (0.69, 1.19)	34	0.73 (0.46, 1.14)		86	0.99 (0.72, 1.35)	58	0.70 (0.49, 0.99)	
	5	106	0.92 (0.69, 1.22)	26	0.55 (0.32, 0.93)		77	0.92 (0.66, 1.28)	53	0.67 (0.46, 0.98)	
	*P*-trend		0.47		0.004			0.58		0.01	
Traditional Australian	1	84	Reference	24	Reference	0.66	69	Reference	36	Reference	0.41
	2	111	1.24 (0.90, 1.71)	42	1.63 (0.85, 3.12)		83	1.22 (0.85, 1.76)	70	1.62 (0.98, 2.66)	
	3	115	1.21 (0.86, 1.70)	39	1.41 (0.71, 2.81)		85	1.28 (0.86, 1.89)	70	1.38 (0.83, 2.32)	
	4	139	1.42 (0.99, 2.03)	55	1.97 (0.98, 3.96)		99	1.52 (1.00, 2.32)	95	1.73 (1.03, 2.92)	
	5	126	1.19 (0.81, 1.74)	42	1.51 (0.69, 3.33)		90	1.40 (0.89, 2.19)	78	1.25 (0.71, 2.21)	
	*P*-trend		0.39		0.34			0.11		0.73	
Meat	1	116	Reference	39	Reference	0.20	86	Reference	69	Reference	0.96
	2	114	1.03 (0.79, 1.34)	38	0.91 (0.58, 1.43)		89	1.07 (0.79, 1.45)	63	0.91 (0.64, 1.28)	
	3	112	1.05 (0.80, 1.37)	47	1.09 (0.70, 1.71)		89	1.09 (0.80, 1.48)	70	1.03 (0.73, 1.45)	
	4	125	1.23 (0.93, 1.62)	40	0.91 (0.56, 1.48)		85	1.10 (0.79, 1.53)	81	1.20 (0.84, 1.71)	
	5	108	1.21 (0.88, 1.66)	38	0.86 (0.50, 1.47)		77	1.13 (0.78, 1.64)	66	1.04 (0.69, 1.55)	
	*P*-trend		0.19		0.48			0.60		0.69	

Abbreviations: ER=oestrogen receptor; PR=progesterone receptor; HRT=hormone replacement therapy.

Estimates from the model including four factor scores, adjusted for country of birth, age at menarche, parity, duration of lactation, oral contraceptive use, HRT use, menopausal status at baseline, physical activity, alcohol, smoking, level of education, total energy intake and BMI.

a Test for homogeneity by ER or PR status.
